# The role of tobacco promoting and restraining factors in smoking intentions among Ghanaian youth

**DOI:** 10.1186/1471-2458-12-662

**Published:** 2012-08-15

**Authors:** David Doku, Susanna Raisamo, Nora Wiium

**Affiliations:** 1 Department of Population and Health, University of Cape Coast, Private Mail Bag, University Post Office, Cape Coast, Ghana; 2 School of Health Sciences, University of Tampere, FI-33014, Tampere, Finland; 3 Centre for Health Promotion, Faculty of Psychology, University of Bergen, Bergen, Norway

**Keywords:** Youth, Smoking, Tobacco use, Smoking Intentions, Ghana

## Abstract

**Background:**

In Western countries, the relationship between smoking intentions and smoking behaviour is well established. However, youth smoking intentions and associated factors in developing countries are largely unexplored and the former may occur for a variety of reasons. We investigated youth smoking intentions in Ghana with regard to several tobacco promoting and restraining factors, including environmental, familial, attitudinal and knowledge measures.

**Methods:**

A school-based survey of a representative sample of 12-20-year-olds was conducted in 2008 in Ghana (*N =* 1338, response rate 89.7%).

**Results:**

In a bivariate model, both among ever and never smokers, allowing smoking on school compound, exposure to tobacco advertisement and parental smoking were associated with future intention to smoke. Compared to those who agreed that smoking is harmful to health, smoking is difficult to quit and that tobacco should not be sold to minors, those who disagreed or were not sure were more likely to have an intention to smoke. In the multivariate analyses, these associations persisted, except that the attitude measures concerning the difficulty of quitting smoking once started and tobacco sales ban were no longer significantly associated with smoking intentions.

**Conclusions:**

These findings underscore the importance of school smoking policy, parental smoking behaviour and knowledge of the harmful effects of tobacco use in determining Ghanaian youths’ future smoking intentions. Because current high percentages of smoking intentions may turn into high smoking rates in the future, the introduction of effective tobacco control measures at all levels of society to prevent youth smoking in Ghana may be essential.

## Background

Smoking continues to be a major but preventable cause of death and diseases worldwide [1]. Recent declines in smoking following a range of tobacco control measures have been limited to developed countries while a rise in smoking rates has been observed in several developing countries [2,3]. It is suggested that if major preventive measures are not put in place, smoking-related death and diseases in many of these developing countries will increase rapidly as the number of smokers continues to rise [1]. Indeed, the WHO report estimates that with the steady increase in tobacco use, more than 8 million people worldwide will die each year by the year 2030, 80% of which will occur in developing countries.

The literature on smoking in many developing countries is scant and for Africa, the available literature suggests considerable variation across the different countries [4]. In Ghana, general rates of smoking are suggested to be low, with males smoking more than females [5,6]. In a random sample of 30 regional census enumeration areas, comprising all individuals 14 years of age and above, [7] smoking rates of 8.9% and 0.3% were reported for males and females, respectively. However, the Global Youth Tobacco Survey (GYTS) reported higher rates among young people between ages 13 and 15. The 2006 GYTS revealed that 11.6% of boys and 10.9% of girls in Ghana used a tobacco product, while 9.4% boys and 8.0% girls have ever smoked cigarettes [8]. Higher rates of tobacco use among the youth may point to a rise in future use. Therefore even if the prevalence of tobacco use in African countries is generally low, it offers an opportunity for the control of the epidemic in these countries, the only region where the epidemic seems to be at its initial stages in many countries.

A large body of research has investigated factors that may determine smoking initiation as well as the relationship between smoking intentions and smoking behaviour among adolescents in Western countries [9-12]. It has been shown that intention to smoke in the future predicts both smoking initiation and subsequent smoking [10-12]. However, little is known about various factors behind youth smoking intentions in developing countries. Identifying factors that influence smoking intentions among young people in Ghana may thus be essential for preventing smoking among this vulnerable group.

Social cognition models have been extensively used to examine factors that tend to predict health behaviour, including smoking. In an integrated theoretical model, Fishbein and colleagues put together concepts from several major social cognition models that may promote or prevent the performance of health behaviour [13]. The social cognition models that constitute the integrative model are the theory of reasoned action [14], the social cognitive theory [15] and the health belief model [16]. In line with the integrative model, behavioural intention, skills and environmental constraints are directly related to behaviour. Thus, a strong intention to smoke, the necessary skills to engage in the behaviour and the ability to overcome environmental constraints could facilitate smoking. For intention to smoke, the model postulates three determining factors: attitudes, perceived norm and self-efficacy. Attitude towards smoking reflects the person’s favourable or unfavourable evaluation of smoking behaviour, while perceived norms reflect both the support from important referents to engage in the behaviour or not and the referents’ own engagement in the behaviour. Self-efficacy expresses the individual’s perception of being able to perform the behaviour under a variety of challenging circumstances.

Although, the social cognition models that constitute the integrative model were developed in Western countries and have mostly been studied in Western populations, the theoretical concepts of the model have been applicable also in non-Western countries. Attitudes, perceived norm and self-efficacy have each been found to predict a range of health behaviours such as oral health, food choices, condom use and smoking, among others in several African and Asian countries [17-19]. The predictive power of these theoretical concepts tends to be affected by the context, a finding which is consistent with the assumption of the integrative model that the relationship between the theoretical factors and behaviour may vary across different populations and behaviours. Smith and colleagues [20] found that differences in intentions between Western and non-Western countries may occur for variety of reasons, for example individuals’ attitudes, beliefs and knowledge, socio-environmental and cultural norms as well as several tobacco-related factors (factors that reflect several of the concepts of the integrative model).

From the public health viewpoint of developing countries, a better understanding of the factors influencing youth smoking intentions is essential to identifying the specific risk groups for smoking, allowing tailored preventive programs to be developed. In this paper, we explored smoking intentions among Ghanaian youth aged 12 to 20 years with regard to tobacco promoting and restraining factors, including several environmental, familial, attitudinal and knowledge measures. Because of the collective culture and the age of the participants, familial and environmental measures may be more strongly associated with smoking intentions than attitudes and knowledge.

## Methods

A survey was conducted in 2008 on health behaviours and lifestyles of youth in Junior and Senior High schools in two out of the three zones in Ghana.

### Sample

Thirty schools were randomly sampled in three regions, ten per region, from Eastern (total number of schools in the region = 2924), Greater Accra (total number of schools in the region = 1825) and Volta Regions (total number of schools in the region = 2184). The present study involves youth aged 12-20-year-old students (N = 1338). The sampling was done as follows: First, ten schools were randomly selected so that they comprise of four public Junior High Schools, two private Junior High Schools, three public Senior High Schools and one private Senior High School in each region in order to reflect the school types in Ghana. Second, in each school, all students whose names were found in the class attendance register of the randomly selected classes were eligible to participate in the survey, after the purpose of the studies was explained to the entire class. The final sample size was 1556, which represented a response rate of 89.7%. The reason for non-response was that pupils were absent from school on the day of data collection. Boys comprised approximately 46% of the sample size.

### Data collection

The eight page questionnaire was anonymous and self-administered and was tested with an initial pilot sample of 50 children in three schools. It was designed to exclude any information that will reveal the identity of the participants. To ensure confidentiality, no teacher was present in the classrooms during the survey. One trained supervisor from the research team was assigned to each classroom during the answering. To prevent contamination, the survey commenced simultaneously in all the participating classes in a given school. Participants were asked to drop their questionnaires in an envelope placed in front of the class on completion. The purpose of the survey was again explained to the randomly sampled adolescents, and they were given the right to accept or decline participation. Furthermore, written informed consents were obtained from 18-year-old adolescents and parents of those under 18 years old who voluntarily agreed to participate in the study.

The study protocol was approved by the ethical committee of the Ghana Health Service Research Unit in Accra, Ghana.

### Measures

*Intention to smoke* was measured by the question: “At any time during the next one year (12 months) do you believe you will smoke a cigarette?” The responses were “Definitely not”, “Probably not”, “Probably yes” and “Definitely yes”. For the analyses, this measure was categorized into two: “No” (definitely not, probably not) and “Yes” (probably yes, definitely yes).

*Smoking* was assessed by the question: “Have you ever tried cigarettes or any other tobacco product?” The response options were “No” and “Yes, which product….”. In this study smokers refer to those who answered “Yes” to the above question (excluding those who mentioned smokeless tobacco product).

### Environmental and familial tobacco promoting/restraining factors

*Smoking on school compound*. Respondents were asked whether smoking was allowed on their school compound or not. The response options were, “yes”, “no” or “I don’t know”.

*Taught the harmful effects of smoking*. Two separate questions were used to assess whether adolescents were taught the harmful effects of smoking in school during the present school year and whether any family member had discussed the harmful effects of smoking with them with the response options “yes/no”.

*Refused cigarette sale due to age*. Respondents indicated “yes” or “no” regarding whether or not they had ever been refused cigarette sale due to their age.

*Exposure to tobacco advertisement*. Respondents indicated whether they had seen any tobacco advertisement during the past month from the following options: billboard, cigarette car/van, newspaper, television, internet/email or other sources.

*Parental smoking.* In two separate questions adolescents were asked to indicate whether their fathers or mothers smoke at present, had never smoked, had smoked but had stopped, whether they couldn’t say anything about parental smoking or had no father or mother. Parental smoking was classified into three categories of “none”, “can’t say” and “one or both parents smoke”.

### Knowledge and attitude indicators

Adolescents indicated whether they completely agree, slightly agree, completely disagree, slightly disagree or were not sure about the following statements: “Smoking is harmful to one’s health”, “Tobacco products should not be sold to those less than 18 years of age” and “Smoking is difficult to quit once started”. The responses were categorised as “agree” (completely agree, slightly agree) and “disagree/not sure” (slightly disagree, completely disagree, not sure).

### Statistical analysis

Pearson’s Chi-square tests (two-tailed *p*-values at a statistical significance level of p < 0.05) were used to test the statistical significance of the relations between the studied variables and smoking intentions. Adolescents with incomplete responses were excluded from the analyses. The frequency of missing values for the explanatory variables varied from 3.3 and 6.2%, except for parental smoking which had 16.6% missing values. Factors associated with smoking intentions were studied using logistic regression analyses. First, bivariate analyses were computed (Model 1) for each of the explanatory variables, adjusting for age and gender. Second, in a multivariate model, the independent associations of all the factors that were statistically significant at the bivariate level were studied, adjusting for age and gender (Model 2). In additional to the total sample, stratified analyses were conducted for never smokers. The results were given as odds ratios (OR) and 95% confidence intervals (CI). SPSS package, version 16 (SPSS Inc, Chicago, Illinois) was used for the analyses.

## Results

### Tobacco promoting and restraining factors by smoking intentions

The characteristics of the sample by youths’ smoking intentions are presented in Table 1. Prevalence of ever smoking was 6.6% (8.0% boys and 4.7% girls). Fifty-seven per cent of ever smokers and 21% of never smokers reported having intentions to smoke during the next one year. In findings not presented in tables, 70% of all respondents were taught the harmful effects of smoking during the school year and 72% had discussions at home about the harmful effects of tobacco use. Regarding parental smoking, 3% had at least one smoking parent, while 2% could not say if their parents smoked or not. Only 4% reported that smoking was allowed on their school compounds. Among those who tried purchasing tobacco products, 43% were not refused because of their age. About half of the youth were exposed to at least one form of tobacco advertisement during the past one month prior to the survey. Majority of adolescents agreed with the statements that “smoking is harmful to one’s health” (83%), “smoking is difficult to quit once started” (74%), and that “tobacco products should not be sold to those less than 18 years of age” (83%).

**Table 1:** Distribution (%) of tobacco promoting and restraining factors among Ghanaian youth by smoking intentions

**Indicators**	**Smoking intentions**		**χ2**	**df**	***P-value (2-tailed)***
	**(N = 971)**	**(N = 299)**			
	**No (%)**	**Yes (%)**			
**Smoking status**			55.2	1	<0.001
Ever smokers	43	57			
Never smokers	79	21			
**Gender**			0.0	1	0.987
Boys	77	23			
Girls	77	23			
**Age**			1.4	3	0.707
12-14 years	75	25			
15-16 years	77	23			
17-18 years	78	22			
19-20 years	74	26			
**Environmental and familial indicators**					
Smoking allowed on school compound			18.9	2	<0.001
Yes	56.2	43.8			
No	78.2	21.8			
Don’t know	60.9	39.1			
Taught harmful effects of smoking in school			4.3	2	0.119
Yes	78.0	22.0			
No	76.3	23.7			
Not sure	67.6	32.4			
Refused cigarette sale due to age			1.8	2	0.185
I never tried to buy tobacco	79.7	20.3			
Yes	49.3	50.7			
No	59.8	40.2			
Exposure to tobacco advertisement			13.4	1	<0.001
Yes	72.3	27.7			
No	81.0	19.0			
Parental smoking			22.3	2	<0.001
None	79.2	20.8			
One or both parents smokes	50.0	50.0			
Can’t say	55.0	45.0			
Family member discussed harmful effects of smoking			1.1	1	0.301
Yes	77.6	22.4			
No	74.8	25.2			
**Knowledge/attitude indicators**					
Perceive smoking as harmful to health			87.7	4	<0.001
Completely agree	81.4	18.6			
Slightly agree	48.6	51.4			
Completely disagree	50.0	50.0			
Slightly disagree	63.2	36.8			
Not sure	44.4	55.6			
It is difficult to quit smoking once started			19.2	4	0.001
Completely agree	50.0	50.0			
Slightly agree	80.3	19.7			
Completely disagree	70.4	29.6			
Slightly disagree	65.8	34.2			
Not sure	68.4	31.6			
Tobacco should not be sold to those under 18 yrs of age			50.0	4	<0.001
Completely agree	80.6	19.4			
Slightly agree	63.5	36.5			
Completely disagree	63.5	36.5			
Slightly disagree	44.8	55.2			
Not sure	61.4	38.6			

Schools that allowed smoking on their compounds registered a higher percentage of students (43.8%) having future intentions to smoke compared to schools that did not (21.8%). Similarly, a higher percentage of respondents who were exposed to tobacco advertisement compared to those who were not reported intentions to smoke in the future (27.7% and 19%, respectively). Also, 50% of respondents with one or both parents who were smokers intended to smoke in the future compared to 20.8% of those whose parents were not smokers. A greater proportion of respondents (55.6%) who were not sure about the harmful effects of smoking, 50% of those who completely agreed that it was difficult to quit smoking once started and 55.2% of those who slightly disagreed that tobacco should not be sold to those under 18 years intended to smoke in the future (see Table 1 for details on the findings).

### Bivariate and multivariate logistic regression analyses of factors associated with smoking intention

Results of the bivariate and multivariate logistic regression analyses are presented in Tables 2 and 3. In a bivariate analyses, after controlling for age and gender (Table 2, Model 1), all the following environmental and familial tobacco promoting/restraining factors were related to smoking intentions among both ever smokers and never smokers: allowing smoking on school compound, exposure to tobacco advertisement and parental smoking. In both the total sample and never smokers’ sample, young people who reported that smoking was not banned on their school compounds were more likely to have intentions to smoke compared to those who reported that smoking was banned on their school compounds (OR = 3.2, 95% CI: 1.7-5.8) and (OR = 3.0, 95% CI: 1.5-6.2), respectively. Similarly, the probability of smoking intentions increased if a youth was exposed to tobacco advertisement, had a parent who smoked or could not say if the parents smoked compared to those who were not exposed to tobacco advertisement and those whose parents were non-smokers. Furthermore, the likelihood of having intentions to smoke increased among those who disagreed or were not sure about the statements that smoking is harmful to health, smoking is difficult to quit once started and tobacco should not be sold to those under 18 year of age, compared to those who agreed (see Table 2, Model 1).

**Table 2:** Odds ratios (OR) and 95% confidence intervals (CI) for smoking intentions by tobacco promoting/restraining factors among Ghanaian youth (bivariate model), statistically significant odds ratios in bold

***Factors***	Smoking intentions*		
	**All participants**	**Never smokers**	
	**Model 1 OR (95% CI)**	**Model 1 OR (95% CI)**	R-squared**
Age			0.002
12-14-year-olds	1.0	1.0	
15-16-year-olds	0.9 (0.6-1.3)	0.9 (0.6-1.3)	
17-18-year-olds	0.8 (0.6-1.2)	0.8 (0.5-1.2)	
19-20-year-olds	1.0 (0.6-1.7)	1.2 (0.7-2.0)	
Gender			0.000
Boys	1.0	1.0	
Girls	1.0 (0.8-1.3)	1.0 (0.8-1.4)	
**Environmental and familial tobacco promoting /restraining factors**			
			
Smoking allowed on school compound			0.09
No (N = 1165)	1.0	1.0	
Yes (N = 50)	**3.2 (1.7-5.8)**	**3.0 (1.5-6.2)**	
Don’t know (N = 48)	**1.9 (1.1-3.8)**	2.0 (0.9-4.2)	
Total (N = 1263)			
Taught harmful effects of smoking in school			
Yes (N = 891)	1.0	1.0	
No/Not sure (N = 372)	1.2 (0.8-1.5)	1.0 (0.9-1.1)	
Total (N = 1263)			
Refused cigarette sale due to age			
No (N = 71)	1.0	1.0	
Yes (N = 93)	1.5 (0.8-2.9)	0.7 (0.3-1.8)	
Total (N = 164)			
Exposure to advertisement			0.08
No (N = 637)	1.0	1.0	
Yes (N = 701)	**1.6 (1.2-2.1)**	**1.5 (1.1-2.1)**	
Total (N = 1338)			
Parental smoking			0.08
None (N = 1063)	1.0	1.0	
Can’t say (N = 21)	**3.1 (1.2-8.0)**	**4.3 (1.2-15.2)**	
One or both parents smoke (N = 36)	**3.7 (1.8-7.6)**	**3.0 (1.4-6.6)**	
Total (N = 1120)			
Family member discussed harmful effects of smoking			
Yes (N = 918)	1.0	1.0	
No (N = 355)	1.1 (0.8-1.5)	1.1 (0.8-1.6)	
Total (N = 1273)			
**Knowledge/attitude**			
Perceive smoking as harmful to health			0.08
Agree (N = 1127)	1.0	1.0	
Disagree/not sure (N = 178)	**3.4 (2.4-4.8)**	**2.9 (1.9-4.3)**	
Total (N = 1305)			
It is difficult to quit smoking, once started			0.08
Agree (N = 971)	1.0	10	
Disagree/not sure (N = 337)	**1.7 (1.2-2.2)**	**1.5 (1.1-2.1)**	
Total (N = 1308)			
Tobacco should not be sold to those under 18 yrs of age			0.08
Agree (N = 1087)	1.0	1.0	
Disagree/not sure (N = 213)	**2.5 (1.8-3.5)**	**2.1 (1.5-3.1)**	
Total (N = 1300)			

*Smoking intentions in the next one year.

** Nagelkerke R-squared.

Model 1 = Factor + age + gender (bivariate model).

**Table 3:** Odds ratios (OR) and 95% confidence intervals (CI) for smoking intentions by tobacco promoting/restraining factors among Ghanaian youth (multivariate model), statistically significant odds ratios in bold

***Factors***	Smoking intentions*		
	**All participants**	**Never smokers**	
	**Model 2 OR (95% CI)**	**Model 2 OR (95% CI)**	R-squared**
**Environmental and familial tobacco promoting /restraining factors**			
			
Smoking allowed on school compound			0.07
No (N = 1165)	1.0	1.0	
Yes (N = 50)	**3.5 (1.6-7.8)**	**4.7 (1.8-11.8)**	
Don’t know (N = 48)	1.0 (0.4-2.4)	1.2 (0.4-3.0)	
Total (N = 1263)			
Taught harmful effects of smoking in school	***	***	
Refused cigarette sale due to age	***	***	
Exposure to advertisement			0.06
No (N = 637)	1.0	1.0	
Yes (N = 701)	**1.4 (1.0-1.9)**	**1.4 (1.0-2.0)**	
Total (N = 1338)			
Parental smoking			0.06
None (N = 1063)	1.0	1.0	
Can’t say (N = 21)	2.4 (0.9-6.4)	**4.7 (1.3-16.9)**	
One or both parents smoke (N = 36)	**3.1 (1.5-6.7)**	**2.4 (1.1-5.7)**	
Total (N = 1120)			
Family member discussed harmful effects of smoking	***	***	
**Knowledge/attitude**			
Perceive smoking as harmful to health			0.06
Agree (N = 1127)	1.0	1.0	
Disagree/not sure (N = 178)	**2.5 (1.5-3.9)**	**2.2 (1.3-3.7)**	
Total (N = 1305)			
It is difficult to quit smoking, once started			0.06
Agree (N = 971)	1.0	1.0	
Disagree/not sure (N = 337)	1.2 (0.7-1.6)	1.1 (0.7-1.7)	
Total (N = 1308)			
Tobacco should not be sold to those under 18 yrs of age			0.06
Agree (N = 1087)	1.0	1.0	
Disagree/not sure (N = 213)	1.2 (0.8-1.9)	1.3 (0.8-2.1)	
Total (N = 1300)			

*Smoking intentions in the next one year.

** Nagelkerke R-squared.

***Not included in the model because variable not statistically significant at the bivariate level (model 1).

Model 2 = All statistically significant factors in model 1 + age + gender + (multivariate model).

In the multivariate model (Table 3, Model 2), the following tobacco promoting and restraining factors were independently associated with smoking intentions in both the total sample and never smokers’ sample: smoking restriction on school compound, exposure to tobacco advertisement, parental smoking and perceptions that smoking is harmful to health. The association of exposure to tobacco advertisement in both samples was however, at borderline level of significance (OR = 1.4, 95% CI: 1.0-1.9) and (OR = 1.4, 95% CI: 1.0-2.0), respectively.

## Discussion

In this study, among both ever and never smokers, allowing smoking on school compound, exposure to tobacco advertisement and parental smoking were all significantly associated with an intention to smoke. In addition, compared to those who agreed that smoking is harmful to health, smoking is difficult to quit and that tobacco should not be sold to minors, those who disagreed or were not sure were more likely to have an intention to smoke in the future. In the multivariate analyses, these associations persisted, except that the attitude measures concerning the difficulty of quitting smoking, once started, and tobacco sales ban to minors were no longer significantly associated with smoking intentions.

On the whole, factors associated with youth smoking intentions in Ghana and Western countries are fairly similar. Previous studies have shown that strongly enforced smoking bans in schools have a protective effect on adolescents’ future smoking [21]. We found that if smoking was allowed on a school compound, youth had a higher intention to smoke than those whose schools banned smoking. An interesting finding in this study was also that among both ever and never smokers, the probability of future smoking intentions was higher among those who disagreed or were not sure regarding the statement that smoking is harmful to one’s health. This finding is consistent with the health belief model [16] which postulates that people’s engagement in behaviours is motivated by their perceived health consequences of the behaviour. Given that the association remained independent of the other tobacco promoting and restraining factors examined in this study suggests that among Ghanaian youth, the belief about the negative health consequences of tobacco use is an important predictor of tobacco use. In view of that, health promotion interventions that would focus on the health damaging effects of tobacco use will be likely to deter the youth from its use in the future.

The difficulty of quitting smoking once started was associated with smoking intentions in bivariate analyses, although not in multivariate analyses. This association to some extent may reflect the influence of self-efficacy in the uptake of the behaviour [13,15], confirming that also among Ghanaian youth self-efficacy could be related to intentions to smoke. Previous studies have found that self-efficacy was associated with health behaviours and behavioural intentions such as intended contraception use among adolescents in Ethiopia [17] and sugar consumption among Ugandan adolescents [18]. Attitude towards a given behaviour has been documented to be an important predictor of intentions [22,23]. In the present study, this finding was confirmed as a youth’s attitude concerning the harmful effects of tobacco use was associated with the probability of intentions to smoke. However, ban on the sale of tobacco products to minors and difficulty in quitting smoking, once started, were not associated with intentions to smoke in multivariate analyses. It is possible that other factors other than attitude also play role in predicting the future smoking intentions. Furthermore, among both ever and never smokers, exposure to tobacco advertisement was associated with smoking intentions, although at borderline level of significance. Advertisement exerts a normative influence on adolescents [13], enhances positive attitude towards smoking and intentions to smoke [24]. Among German adolescents exposure to cigarette ads, but not other adverts, was found to increase the chances of intentions to smoke [24].

Consistent with the social cognitive theory [15], parental smoking was found to be significantly predictive of intentions to smoke such that adolescents with at least one smoking parent were at higher risk of smoking in the future compared to those without smoking parents. This association was independent of the other predictive factors investigated in the present study. In the United Kingdom, parental smoking was related with youths’ smoking intentions [25]. The relationship between parental smoking and their children’s smoking uptake has been well documented [9,26]. In addition to the role modelling plausibility of the impact of parental smoking on adolescent smoking, children with smoking parents may not only consider the behaviour as a norm but may also have access to cigarettes at home [26] and thus be more likely to initiate and maintain the behaviour compared to those with non-smoking parents. It is likely that the same mechanisms underline the relationship between parental smoking and the intention to smoke found in the present study.

### Strengths and limitations

This study was the first of its kind to be conducted among Ghanaian youth. We used a representative sample of schools in three regions in Ghana which are representative of the entire country. The overall response rate was high (89.7%). This study therefore fills an important gap in literature in both Ghana and in developing countries as a whole with respect to factors associated with smoking intentions. Despite these strengths, the study has some limitations. As the sample of students was drawn from a sample of schools, the clustering of students may slightly change the standard error of our estimates, although unlikely to change neither the overall results nor the conclusion reached. The low prevalence of smoking among Ghanaian adolescents could not allow for the use of other categorisation of smoking other than “ever smoking”. A limitation of this classification of smoking is that it includes ex-smokers as well as those who have had just a puff. Despite this limitation, the results of this study in general, provide an overview of smoking and smoking intentions among Ghanaian adolescents. The listwise deletion of missing data employed in the handling of missing data may lead to bias, especially where there are large missing data. However, as missing data was low in this study (<5% for most variables), the listwise deletion of missing data is unlikely to affect the estimates or alter the conclusions reached. We acknowledged some limitations that are mainly related to self-reporting in a cross-sectional study design. Thus, the cause and effect relationship cannot be highlighted. In addition, since all data were based on self-reports, we cannot exclude the possibility of under- or over-reporting.

## Conclusions

A number of tobacco promoting and restraining factors including, allowing smoking on school compound, exposure to tobacco advertisement, parental smoking and perception that smoking is harmful to one’s health were associated to intentions to smoke among Ghanaian youth. Thus, to a large extent, the present study supports the integrated theoretical model, particularly, the associations of normative processes and attitude with intentions to smoke. The importance of school smoking policy, parental smoking behaviour and knowledge of the harmful effects of tobacco use in determining Ghanaian youths’ future smoking intentions is emphasised. Current smoking rates in Ghana may be low. Nevertheless, the present study indicates a probable increase in future rates as smoking intentions appear to be considerably high among smokers and non-smokers alike. Despite the ban on all cigarette advertisements on national television and radio, tobacco control measures in Ghana still fall short of several effective strategies recommended by the World Health Organization. “Raising taxes and prices, banning advertising, promotion and sponsorship, protecting people from second-hand smoke, warning everyone about the dangers of tobacco, offering help to people who want to quit, and carefully monitoring the epidemic and prevention policies” are six WHO tobacco control strategies that are not in place in Ghana yet, probably because of the low smoking rates. However, because the relatively high percentages in current smoking intentions may turn into high smoking rates in the future, a ban on cigarette advertisements may not be sufficient. The introduction of more effective tobacco control measures at all levels of society to prevent youth smoking may be essential.

## Competing interests

The authors declare that they have no competing interests.

## Authors’ contributions

DD, NW and SR were involved in the conception and design of the study. DD and SR were involved in the drafting and the revising of the questionnaire. DD was the principal investigator during the data collection. DD analysed the data and drafted the manuscript. All authors were involved in the interpretation of data and the critical revision of the manuscript for important intellectual content. All authors gave final approval of the version to be published.

## Funding

The study was financially supported by the Finnish Cultural Foundation Central Fund, Tampere University research stipend and the Juho Vainio Foundation

## Pre-publication history

The pre-publication history for this paper can be accessed here:

http://www.biomedcentral.com/1471-2458/12/662/prepub
